# Melatonin Therapy Improves Cardiac Autonomic Modulation in Pinealectomized Patients

**DOI:** 10.3389/fendo.2020.00239

**Published:** 2020-04-30

**Authors:** Luciana Aparecida Campos, Clarissa Bueno, Isabella P. Barcelos, Bruno Halpern, Leandro C. Brito, Fernanda G. Amaral, Ovidiu Constantin Baltatu, José Cipolla-Neto

**Affiliations:** ^1^Center of Innovation, Technology and Education (CITE) at São José dos Campos Technology Park, São Paulo, Brazil; ^2^Institute of Biomedical Engineering, Anhembi Morumbi University, Laureate International Universities, São José dos Campos, Brazil; ^3^College of Health Sciences, Abu Dhabi University, Abu Dhabi, United Arab Emirates; ^4^Department of Physiology and Biophysics, Institute of Biomedical Sciences, University of São Paulo, São Paulo, Brazil; ^5^Department of Pediatric Neurology, Hospital das Clínicas of University of São Paulo Medical School, São Paulo, Brazil; ^6^Department of Endocrinology and Metabolism, Hospital das Clínicas of University of São Paulo Medical School, São Paulo, Brazil; ^7^Exercise Hemodynamic Laboratory, School of Physical Education and Sport, University of São Paulo, São Paulo, Brazil; ^8^Department of Physiology, Federal University of São Paulo, São Paulo, Brazil; ^9^Department of Pharmacology and Therapeutics, College of Medicine & Health Sciences, Khalifa University, Abu Dhabi, United Arab Emirates

**Keywords:** melatonin, therapeutics, pinealectomy, pineal tumor, heart rate variability

## Abstract

The purpose of this investigational study was to assess the effects of melatonin replacement therapy on cardiac autonomic modulation in pinealectomized patients. This was an open-label, single-arm, single-center, proof-of-concept study consisting of a screening period, a 3-month treatment period with melatonin (3 mg/day), and a 6-month washout period. The cardiac autonomic function was determined through heart rate variability (HRV) measures during polysomnography. Pinealectomized patients (*n* = 5) with confirmed absence of melatonin were included in this study. Melatonin treatment increased vagal-dominated HRV indices including root mean square of the successive R-R interval differences (RMSSD) (39.7 ms, 95% CI 2.0–77.4, *p* = 0.04), percentage of successive R-R intervals that differ by more than 50 ms (pNN50) (17.1%, 95% CI 9.1–25.1, *p* = 0.003), absolute power of the high-frequency band (HF power) (1,390 ms^2^, 95% CI 511.9–2,267, *p* = 0.01), and sympathetic HRV indices like standard deviation of normal R-R wave interval (SDNN) (57.6 ms, 95% CI 15.2–100.0, *p* = 0.02), and absolute power of the low-frequency band (LF power) (4,592 ms^2^, 95% CI 895.6–8,288, *p* = 0.03). These HRV indices returned to pretreatment values when melatonin treatment was discontinued. The HRV entropy-based regularity parameters were not altered in this study, suggesting that there were no significant alterations of the REM–NREM ratios between the time stages of the study. These data show that 3 months of melatonin treatment may induce an improvement in cardiac autonomic modulation in melatonin-non-proficient patients.

**ClinicalTrials.gov Identifier:** NCT03885258.

## Introduction

Melatonin, produced by the pineal gland, following a circadian rhythmic profile peaking during the night, is considered as a major hormone regulating the circadian rhythmicity of biological systems (1, 2). Melatonin has been implicated in several regulatory functions of the cardiovascular system (3, 4). Accumulating evidence indicates that melatonin plays a protective role in various cardiovascular diseases (5), mainly due to its antioxidative, antiapoptotic, and anti-inflammatory actions (6). Since experimental pinealectomy was shown to induce an increase of blood pressure (7), one hypothetical pharmacological use of melatonin is in lowering hypertension (8, 9). Melatonin's actions are mediated through interaction with specific MT1 (or MTNR1A) and MT2 (or MTNR1B) receptors (G-protein coupled membrane-bound melatonin receptors), the quinone reductase II enzyme (previously defined the MT3 receptor), or with nuclear orphan receptors from the RORα/RZR family (10). MT1 and MT2 receptors are found in cardiovascular organs including peripheral and central blood vessels, heart, kidneys, and adrenal glands. Stimulation of vascular melatonin receptors induces vasoconstriction (MT1) or vasodilation (MT2) depending on its concentration (11, 12).

We previously indicated in a mechanistic proof-of-concept preclinical study, using an experimental model of area postrema ablation, that melatonin may modulate baroreceptor reflex control of heart rate via melatonin receptors in the area postrema, which is located outside the blood–brain barrier (13). This study indicates a mechanism for the melatonin interactions with cardiac autonomic function. Indeed, other studies have shown that melatonin modulates the tone of the autonomic nervous system (14). This is why we hypothesized that melatonin replacement therapy in pinealectomized patients may affect cardiac autonomic function.

The purpose of this clinical proof-of-concept study was to investigate whether melatonin replacement therapy may affect cardiac autonomic function in patients showing absence of circulating melatonin as a consequence of pinealectomy due to pineal tumors.

## Methods

This was an open-label, single-arm, single-center, proof-of-concept study to assess the effects of melatonin on cardiac autonomic modulation in melatonin-non-proficient pinealectomized patients. The study consisted of a screening period, followed by a 3-month melatonin treatment period, and a 6-month washout follow-up period. Adverse events were monitored with respect to seriousness, intensity, relationship to treatment, action taken, and outcome of the event.

The protocol was approved by the University of São Paulo Ethical Committee (30460114.5.0000.0068) and was conducted in accordance with International Council for Harmonization of Technical Requirements for Pharmaceuticals for Human Use Guidelines and the Declaration of Helsinki. All patients or patients' parents/legal guardians provided written informed consent before entry into the study. The polysomnography study was performed at the Children's Institute of the Clinical Hospital of the University of São Paulo.

### Patient Selection

Inclusion criteria were patients with pinealectomy and all of the following criteria were considered for admission to the clinical trial: children, adolescents, and young adults 0 months−25 years of age; signed written informed consent (patient or his/her parents/legal guardian); willing and able to complete the clinical trial procedures, as described in the protocol. Key eligibility criteria were no recurring brain tumor after pineal ablation and subsequent chemotherapy and absence of circulating melatonin evaluated by salivary melatonin ELISA assay (IBL International, Hamburg, Germany). All subjects included in the present study had confirmed absence or very low (below 5 pg/ml) non-fluctuating levels of salivary melatonin measured every 3 h for consecutive 27 h.

Exclusion criteria were patients with cardiac arrhythmias, patients with visual loss, and potentially non-compliant subjects (patients who had not completed one of the phases of the study or who did not present salivary melatonin levels >5 pg/ml during the dark phase after melatonin replacement).

We identified six patients in our hospital who submitted to pinealectomy and currently in follow-up. All patients started the clinical trial, but only five completed the protocol. The sixth patient refused to have the polysomnography performed after melatonin introduction and was excluded.

### Melatonin and Polysomnography Measurements

**Figure 1:**
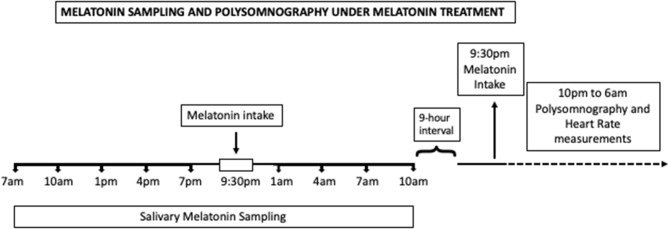
Timeline of the study under melatonin treatment. Melatonin intake time range is represented by a white bar during the sampling night. During the polysomnography night, melatonin was administered at 9:30 p.m., represented by the black arrow.

A two-night monitoring was performed in the Children's Institute of the Clinical Hospital of the University of São Paulo. Sampling of salivary melatonin was performed in the first night, and the polysomnography with ECG measurements were performed in the second night in two conditions: before and after 3 months of melatonin treatment (Figure 1).

Salivary melatonin sampling was performed in a fixed schedule, every 3 h, along 27 h, from 7 p.m. until 10 p.m. the next day, under environmental dim light condition (Supplementary Figure 1). During the sampling night, patients were woken up every 3 h for the procedure. The sampling of salivary melatonin was also performed under melatonin treatment in the night before the polysomnography (Figure 1), according to the same protocol detailed above. During the melatonin sampling night, patients were instructed to take melatonin 30 min before their usual bedtime that was around 10 p.m.

Polysomnography recordings started around 10 p.m. and finished around 6 a.m. according to the institutional protocol. Those recordings were used for heart rate variability (HRV) evaluation. During the polysomnography night under melatonin treatment, all patients received melatonin inside the hospital at 9:30 p.m.

Salivary melatonin was measured by direct saliva ELISA method (IBL International, Hamburg, Germany). Values below 0.3 pg/ml were stated as non-detectable (ND) as this is the analytical sensitivity of the used ELISA kit. Melatonin was measured under environmental dim light condition during the night and indoor light during the day, according to light exposition in daily life. Patients used an actigraphy with light sensor during this period, and the average light levels during the night were 11 ±10 lux. We defined the night interval from 7 p.m. (when saliva sampling started) to 5 a.m. (as the last sample before daily activities start would have been collected at 4 a.m.), to calculate the average night illuminance, for all subjects. Subjects were asked to keep the actigraphy device uncovered during the day and night recordings. The rationale to use samples chosen ~3–6 h into the sleep episode was to investigate the endogenous melatonin profile across the night after melatonin supplementation, once it was a melatonin-depleted population whose levels along the whole night would depend exclusively on the exogenous melatonin.

### Melatonin Replacement Therapy

The dosing regimen was derived from previous studies reporting clinical efficacy on reported outcomes following oral administration (2, 15, 16). Melatonin (Aché Pharmaceutics, Brazil), 3 mg, was administered in the evening, 30 min before the usual bedtime (determined after patient interview, sleep log and actigraphy recording analysis in the previous month to melatonin administration), every day for 3 months. Bedtime was defined according to sleep diary recording, and the actigraphy software was then used to calculate estimated actual time asleep (Supplementary Figure 1). The usual bedtime that was around 10 p.m. The calculation was performed considering only the time frame between sleep diary-based bedtime and sleep diary-based out-of-bed time. Although one of the subjects had sleep duration up to 10 a.m., it can still be considered in the range of interindividual variability. This specific patient had no labor or study activities when the study was performed, which possibly allowed him to have longer sleep duration. After discontinuation of melatonin therapy, patients were followed up for 6 months to assess safety parameters and cardiac autonomic modulation.

### ECG Measurement and Heart Rate Variability Analysis

The cardiac autonomic function was determined through HRV measures after polysomnography ECG recordings. Polysomnography recordings started around 10 p.m. and finished around 6 a.m. according to the institutional protocol of the Children's Institute of the Clinical Hospital of the University of São Paulo, where the study has been performed. Patients had previous blood pressure measures in normal ranges (90–120/60–80 mm Hg).

The sampling rate of the ECG signal was 200 Hz (5-ms resolution). The system automatically filters all artifacts and ectopic beats and generates a regular signal by linear interpolation of the heart rate tachogram. The duration of the recordings was 480 min. HRV was assessed with the Kubios HRV analysis software (Department of Applied Physics, University of Eastern Finland) (17). Correction of artifacts in R-R interval data before HRV analysis was through the Kubios's automatic correction algorithm. Cardiac autonomic modulation was determined through time-domain, frequency-domain, and non-linear measures of HRV (18, 19).

Time-domain measures included: standard deviation of normal R-R wave interval (SDNN), root mean square of the successive R-R interval differences (RMSSD), pNN50 (percentage of successive R-R intervals that differ by more than 50 ms), and triangular index (integral of the density of the R-R interval histogram divided by its height).

Frequency domain measures obtained through autoregressive (AR) modeling included the absolute power of the low-frequency band (LF power) (0.04–0.15 Hz), absolute power of the high-frequency band (HF power) (0.15–0.4 Hz), and LF/HF ratio.

Non-linear measures were Poincaré plot standard deviation perpendicular the line of identity (SD1), Poincaré plot standard deviation along the line of identity (SD2), and ratio of SD1 to SD2 (SD1/SD2).

### Statistical Analysis

Descriptive statistics included mean, standard error for symmetrically distributed continuous variables. Kolmogorov–Smirnov test, with the Dallal–Wilkinson–Lilliefor corrected *P*-value was used to test normality distribution since it can calculate a minimum of five values.

For continuous end points, the changes between pretreatment baseline, treatment, and follow-up were assessed with repeated-measures one-way ANOVA with Geisser–Greenhouse correction followed by Tukey test for multiple comparisons. Within-patient changes were used for efficacy comparisons. *P*-values presented are two-sided and are considered significant at the 0.05 level. All statistical analyses were carried out using GraphPad Prism version 6.0e for Mac OS X, GraphPad Software, La Jolla, California, USA, www.graphpad.com. Differences were considered significant when the probability of a Type I error was lower than 5% (*p* < 0.05).

## Results

Four male and one female patients (age range 12–21) with pinealectomy were included in this study. No postoperative measurable residual pineal tumors were detected, and absence of circulating melatonin was an inclusion criterion. No participant withdrew because of side effects. Table 1 shows the characteristics of study participants.

**Table 1:** Characteristics of study participants.

**Patient**	**Age (years)**	**Type of tumor**	**Years between pinealectomy and melatonin**	**Average sleep onset**	**Average sleep duration**
1	21	Germinoma	2	10:30 p.m.	10 h 00 min
2	19	Pinealoblastoma	7	10 p.m.	7 h 30 min
3	16	Germinoma	7	8:50 p.m.	8 h 50 min
4	21	Germinoma	3	10:10 p.m.	9 h 40 min
5	12	Germinoma	5	00:30 a.m.	8 h 30 min

Patients showed absence of or low secretion of melatonin at all the time points throughout the day, every 3 h on a 27-h schedule, from 7 p.m. until 10 p.m. the next day, under environmental dim light condition (Table 2) (20, 21). Salivary melatonin was also collected just before performing the polysomnography under melatonin treatment (at the end of the third month of treatment), according to the same protocol (every 3 h for consecutive 27 h). Only subjects who exhibited melatonin levels >5 pg/ml ~3.5–6.5 h after melatonin intake (average mean value = 22.29 pg/ml) were included. These two time points were selected because they could reliably be considered as time points after melatonin intake. All patients had sleep duration adequate for age according to actigraphy monitoring and sleep logs recorded during the whole previous month. Although sleep was truncated in the morning for polysomnographic recordings, all patients had sufficient sleep efficiency to allow analysis of sleep architecture, with median sleep efficiency of 84.8% in baseline conditions, 80.1% after melatonin replacement, and 82.75% during washout period. There were no alterations in habitual sleep timing between baseline, during treatment, and after washout.

**Table 2:** Average salivary melatonin (pg/ml) before melatonin treatment.

**Hour**	**Patient 1**	**Patient 2**	**Patient 3**	**Patient 4**	**Patient 5**
7 p.m.	ND	ND	ND	ND	ND
10 p.m.	ND	ND	0.59	ND	ND
1 a.m.	ND	ND	ND	ND	ND
4 a.m.	ND	ND	ND	ND	3.68
7 a.m.	ND	ND	ND	ND	3.68
10 a.m.	ND	ND	ND	ND	1.31
1 p.m.	ND	ND	ND	ND	0.46
4 p.m.	0.59	ND	ND	ND	ND
7 p.m.	ND	ND	ND	ND	ND
10 p.m.	ND	ND	0.78	ND	ND

*ND, non-detectable levels according to the kit analytical sensitivity (0.3 pg/ml)*.

Average levels of heart rate were not significantly different between the several time stages of the study (beats per minute: mean ± SD): 77.9 ± 8.5 (pretreatment baseline), 89.4 ± 12.2 (treatment), and 82.9 ± 12.3 (washout).

**Figure 2:**
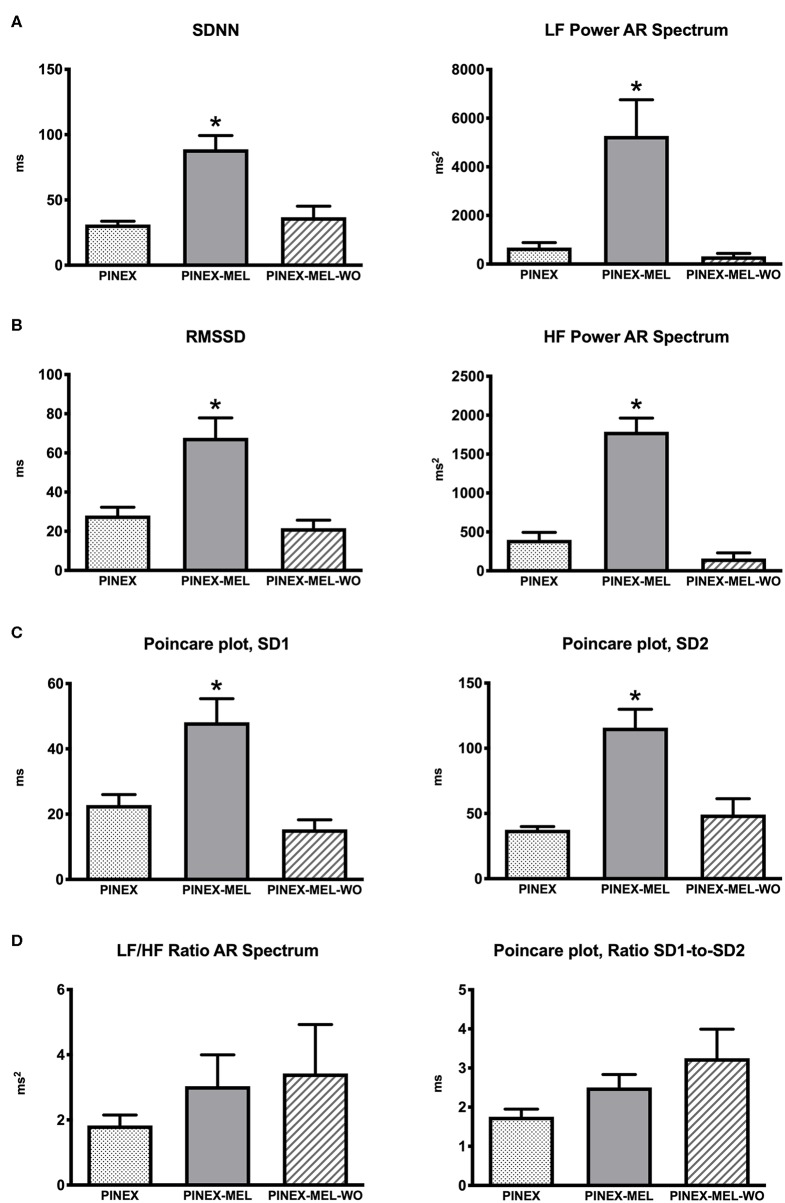
**(A)** Sympathetic modulation heart rate variability (HRV) measures: standard deviation of normal R-R wave interval (SDNN) and absolute power of the low-frequency band (LF power) autoregressive (AR) spectrum; *p < 0.05 significantly different from the other groups. **(B)** Parasympathetic modulation measures: root mean square of the successive R-R interval differences (RMSSD) and absolute power of the high-frequency band (HF power) AR spectrum; *p < 0.05 significantly different from the other groups. **(C)** Parasympathetic modulation: Poincaré plot analysis measures of SD1 and SD2; *p < 0.05 significantly different from the other groups. **(D)** Autonomic balance estimated through the ratio of SD1 to SD2 of Poincaré plot and LF/HF ratio of AR spectrum.

Sympathetic and parasympathetic modulation was significantly increased by melatonin treatment: SDNN (57.6 ms, 95% CI: 15.2–100.0, *p* = 0.02) and LF power (4,592 ms^2^, 95% CI: 895.6–8,288, *p* = 0.03) (Figure 2A). Vagal-dominated HRV indices were increased by melatonin, including RMSSD (39.7 ms, 95% CI: 2.0–77.4, *p* = 0.04) and HF power (1,390 ms^2^, 95% CI: 511.9–2,267, *p* = 0.01) (Figure 2B). Also, melatonin increased the pNN50 (17.1%, 95% CI: 9.1–25.1, *p* = 0.003). Parasympathetic modulation estimated through Poincaré measures was increased by melatonin: SD1 (25.4 ms, 95% CI: 3.9–46.9, *p* = 0.03) and SD2 (78.3 ms, 95% CI: 24.2–132.5, *p* = 0.01) (Figure 2C).

RMSSD and SD1 data provided similar statistical results because they are identical HRV metrics (22, 23). Also, SDNN and SD2 are analogs and provided similar statistical results (24).

Overall HRV estimated by triangular index was increased by melatonin (7.6, 95% CI: 2.2–13.1, *p* = 0.02). After the cessation of melatonin treatment, all HRV indices returned to the values preceding the treatment. Autonomic balance estimated through the SD1/SD2 of Poincaré plot and LF/HF ratio of AR spectrum were not significantly different between the three time phases of the study (Figure 2D).

Finally, approximate entropy and sample entropy analysis used as a measure of complexity were not altered by melatonin treatment and across the study timelines (mean ± SD, approximate entropy: 0.59 ± 0.06, 0.57 ± 0.05, 0.55 ± 0.11; sample entropy: 2.05 ± 0.95, 1.24 ± 0.4, 1.34 ± 0.40).

## Discussion

The findings of the present proof-of-concept study done in humans provide evidence that melatonin replacement therapy in melatonin-deficient patients may induce an improvement in cardiac autonomic modulation in humans. Melatonin treatment increased vagal-dominated HRV indices including RMSSD and also SDNN and LF, reflecting both sympathetic and vagal modulation of heart rate.

Melatonin administration in otherwise melatonin-non-proficient patients induced a drastic increase of HRV indices as measures of autonomic cardiac modulation. Accumulating evidence indicates that autonomic cardiac disfunction is implicated as a pathophysiological link between sleep disorders and their physiological consequences (25–28). Polysomnogram HRV as a measure of cardiac autonomic modulation is a non-invasive, automatable biomarker of cardiovascular risk (29), and is independently associated with carotid atherosclerosis (30). The HRV metrics mostly correlate with clinical improvement (31). Previous studies have demonstrated melatonin administration effects on cardiovascular autonomic function (32, 33) and on relations between sleep electroencephalogram (EEG) and HRV.

In the present study, melatonin replacement therapy significantly augmented the HRV and, thus, the cardiac autonomic function during sleep. Further studies shall investigate the potential pathophysiological link between reduction in melatonin production and cardiac autonomic function.

HRV analysis is a commonly used non-invasive method to measure alterations in autonomic tone with predictive value in diseases. We have identified polysomnography HRV algorithms associated with sleep apnea severity (26). Time-domain, frequency-domain, and non-linear analyses of HRV are reliable approaches to assess changes of autonomic cardiac modulation during sleep both in health and diseases (28, 34). The SDNN reflects both vagal and sympathetic modulation while the RMSSD and pNN50 are indicative of vagal action (35). The SDNN is highly correlated with the LF power (36), which represents an indirect measure of baroreflex function (37, 38). Indeed, bilateral carotid body tumor resection being associated with decreased LF power with no differences in HF power and LF/HF ratio of HRV during sleep indicates that sleep HRV is generated through both baroreflex and central, non-baroreflex-mediated pathways (39). The RMSSD and Poincaré plot analysis measures reflect largely parasympathetic modulation and are less affected by respiratory rate, heart rate, or recording duration (40, 41). High-frequency power is highly correlated with the pNN50 and RMSSD time-domain measures (31).

Improvement in sleep or alterations of the REM–NREM ratios may be considered the major contributor to HRV changes. However, the HRV entropy-based regularity parameters were not altered in this study, indicating no significant alterations of the REM–NREM ratios between the time stages of the study (42). Also, pinealectomy apparently does not cause specific sleep impairment (43). In this study, the median sleep efficiency and habitual sleep timing were not changed between baseline, during treatment, and after washout. Detailed polysomnographic EEG and sleep analysis on pinealectomized patients are underway (44).

This study has some limitations. The greatest limitation of this exploratory study was the small sample size since pineal tumors are rare and thus pinealectomy is a rare procedure. Other study limitations include the open-label, non-randomized design, and absence of a placebo arm. Also, patients were woken up every 3 h for salivary sampling for melatonin measurements. Although it is a fast procedure, sleep fragmentation is a consequence and might have an impact on the following night. However, this was the same protocol used before and after melatonin administration, in such a way that the impact on polysomnography data is expected to be the same in both situations. Another limitation of the present study is that it does not include pharmacokinetics and pharmacodynamics research. Early confirmatory phase studies to support further proof-of-concept such our present study should include phase 1 single ascending dose (SAD), multiple ascending dose (MAD), and absorption, distribution, metabolism, and excretion (ADME) studies (3).

Although this study consisted of only five pinealectomized patients, the drastic increase in HRV by melatonin among the patients sets a precedent for a larger, randomized, placebo-controlled study. Though the present study has limitations, it serves as the first in-human trial to explore the use of melatonin as supportive therapy for patients with pinealectomy. As a proof-of-concept study, these results might be extended to other clinical and/or epidemiological situations where hypomelatoninemia is detected such as aging, diabetes, light at night pollution, and shiftwork (2).

Moreover, to the best of our knowledge, this is the first study of melatonin on cardiac autonomic nervous system in patients with pinealectomy. These data show that 3 months of melatonin treatment may induce an improvement in cardiac autonomic modulation in patients with pinealectomy. Follow-up placebo-controlled clinical trials in diverse patient populations are warranted to further define the role of melatonin in improving autonomic cardiac modulation.

## Data Availability Statement

The datasets generated for this study are available on request to the corresponding author.

## Ethics Statement

The studies involving human participants were reviewed and approved by University of São Paulo Ethical Committee. Written informed consent to participate in this study was provided by the participants' legal guardian/next of kin.

## Author Contributions

JC-N, CB, FA, and OB contributed to study conception and design. CB, IB, and BH performed the study. LC, OB, LB, BH, and FA performed the assays and data analysis. JC-N, OB, and LC contributed to interpretation of the data, writing of the manuscript, and critical revision of the manuscript regarding the important intellectual content.

## Conflict of Interest

The authors declare that the research was conducted in the absence of any commercial or financial relationships that could be construed as a potential conflict of interest.

## Supplementary Material

Supplementary Figure 1 Light intensity during the 27 h of melatonin sampling.
