# Supplementary figures and images for: Melatonin Therapy Improves Cardiac Autonomic Modulation in Pinealectomized Patients
Source: Front Endocrinol (Lausanne). 2020 Apr 30;11:239. doi: 10.3389/fendo.2020.00239 (PMC7213221; doi:10.3389/fendo.2020.00239)

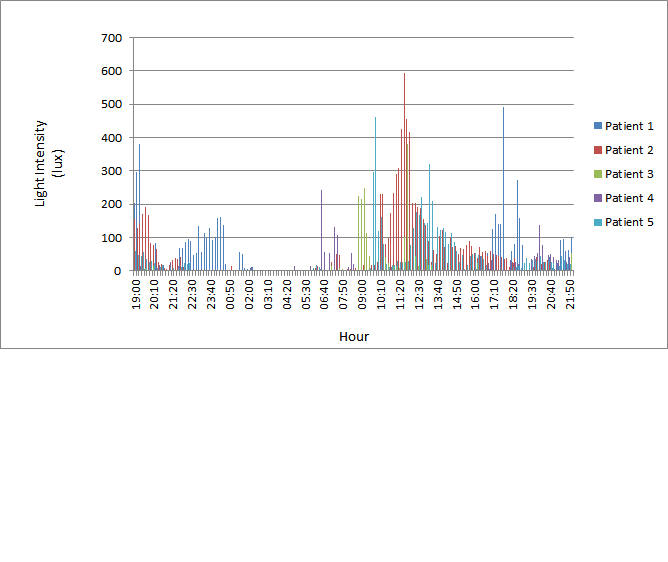

Supplement: Supplementary Figure 1 — Light intensity during the 27 h of melatonin sampling. [file Image_1.tif]
